# Analysis of Fecal Microbiome and Metabolome Changes in Goats When Consuming a Lower-Protein Diet with Varying Energy Levels

**DOI:** 10.3390/microorganisms13040941

**Published:** 2025-04-18

**Authors:** Hu Liu, Anmiao Chen, Wenji Wang, Weishi Peng, Kaiyu Mao, Yuanting Yang, Qun Wu, Meng Zeng, Ke Wang, Jiancheng Han, Hanlin Zhou

**Affiliations:** 1 Zhanjiang Experimental Station, Chinese Academy of Tropical Agricultural Sciences, Zhanjiang 524013, China; liuh2018@lzu.edu.cn (H.L.); cam1835287831@163.com (A.C.); m17378095519@163.com (W.P.); mmiaozzi@163.com (K.M.); ytyang10@163.com (Y.Y.); wuqun.2006@163.com (Q.W.); zmeng0909@163.com (M.Z.); lp-wangke@163.com (K.W.); hanjiancheng810@163.com (J.H.); 2 College of Animal Science and Technology, Guangxi University, Nanning 530004, China; 3 Department of Animal and Veterinary Sciences, AU Viborg-Research Center Foulum, Aarhus University, DK 8830 Tjele, Denmark; wangwj@anivet.au.dk

**Keywords:** goats, microbiome, metabolomics, fecal, lower-protein diet with varying energy levels

## Abstract

The objective of this study was to evaluate the effect of fecal bacterial community and metabolomics in goats when consuming a lower-protein diet with different energy levels. Eight healthy Leizhou goats, with 11 ± 0.78 kg of body weight, were selected and housed individually in cages. The animals were randomly allocated to a lower-protein diet that varied with four metabolites energy levels (7.01, 8.33, 9.66, and 10.98 MJ/kg DM) in a replicated 4 × 4 Latin square design. Notably, energy-dependent microbial restructuring was observed at both phylum and genus levels. At the phylum level, the relative abundances of Firmicutes and Spirochaetote increased linearly, whereas the Bacteroidota and Patescibacteria decreased linearly with increasing dietary energy levels (*p* < 0.05). The relative abundances of Verrucomicrobiota increased quadratically, whereas others decreased quadratically with increasing dietary energy levels (*p* < 0.05). At the genus level, a total of 316 bacteria were identified in the 32 fecal samples. The relative abundances of *Christensenellaceae_R-7_group*, *unclassified_f__Lachnospiraceae*, *Ruminococcus*, *norank_o__Clostridia_UCG-014*, *Treponema*, *[Eubacterium]_siraeum_group*, and *[Eubacterium]_ruminantium_group* increased linearly, whereas the *Oscillospiraceae_UCG-005*, *norank_f__[Eubacterium]_coprostanoligenes_group*, *Prevotellaceae_UCG-004*, *unclassified_c__Clostridia*, *norank_f__Ruminococcaceae*, *unclassified_f__ Oscillospiraceae*, and others decreased linearly with an increasing dietary energy levels (*p* < 0.05). In addition, the metabolomic analysis of feces showed that there are many differential metabolites in goats when consuming a lower-protein diet with different energy levels; for example, lipid metabolism and amino acid metabolic pathways were increased in MLE, MHE, and HE groups compared to the LE group. In conclusion, this study provides further information regarding the effects on fecal bacterial community composition and metabolites in goats when consuming a lower-protein diet with different energy levels.

## 1. Introduction

The Leizhou goat (*Capra hircus*), also named the Hainan Black goat, is the only indigenous goat breed in the Leizhou Peninsula and Hainan Island. As the tropical, well-adapted small ruminant breeds, Leizhou goats were characterized by high-quality meat and adaptability to humid and hot environments. There are more than 1.5 million Leizhou goats, and their production performance, litter sizes, and carcass yields are lower [1].

Dietary nutritional levels play a vital role in animal production, critically influencing growth performance and economic viability. Previous studies reported that a greater energy level could improve the production performance of Boer goats [2], Yunnan semi-fine wool sheep [3], Tan sheep [4], and Hu lambs [5]. Similarly, higher protein levels could also improve the animal performance in Anhui white goat kids [6] and growing Korean Black goats [7]. However, there is little information focused on the dietary energy and protein levels of Leizhou goats. In addition, high-energy and high-protein diets can lead to a waste of feed ingredients. Therefore, it is necessary to determine the optimal energy and protein levels to maximize animal production and achieve the greatest profit.

Currently, the Chinese government emphasizes developing lower-protein diets in animal production, driven by dual imperatives of mitigating nitrogen pollution and addressing protein feedstock shortages. In addition, this strategic shift gains urgency from the escalating economic and environmental costs associated with conventional high-protein diets for animal production. However, many studies have reported the critical role of fecal bacteria, which is closely related to the immunity, digestion, and production performance of ruminants [8,9]. Previous studies reported that the fecal bacterial communities changed in lactating ewes [10] and dairy cows [11] when consuming a lower-protein diet.

By analyzing alterations in the compositional profiles of metabolites within an organism and their associated metabolic pathways, metabolomics offers a comprehensive and insightful approach to elucidating the physiological changes occurring within the organism. It was reported that 125 differential metabolites were screened in positive ion mode and enriched in 12 metabolic pathways, and in negative ion mode, 100 differential metabolites were screened and enriched in 7 metabolic pathways in pregnant goats [12]. Moreover, the fecal metabolite profiles were also reported on sheep [13] and lactating ewes [10] when consuming a lower-protein diet. Dietary energy is a vital factor affecting the rumen bacterial composition in ruminants [14]. However, there is little information on the fecal bacterial and metabolite changes in Leizhou goats when consuming a lower-protein diet with different energy levels.

In this study, we aimed to investigate the fecal bacterial communities and metabolomics profiles by using 16S rRNA sequencing together with untargeted metabolomics in Leizhou goats when consuming low-protein diets with different energy levels. The results provide further information regarding the changes in fecal bacterial communities and metabolism, which may be useful for developing a dietary formulation for optimizing Leizhou goats.

## 2. Materials and Methods

This experiment lasted for 16 weeks, from Jul. to Nov. 2024, and was carried out at the goat farm in Zhanjiang City, Guangdong Province, China. The goat farm belongs to the Zhanjiang Experimental Station (ZES), Chinese Academy of Tropical Agricultural Sciences.

### 2.1. Animals and Experimental Design

A total of 8 healthy Leizhou female goats (8 months, 11 ± 0.78 kg) were used in a replicated 4 × 4 Latin square experimental design, consisting of 4 dietary treatments and 4 periods, with 2 goats per treatment of each period. For each period, an initial 25 d were used for diet adaptation periods, and the final 3 d were used for sampling and data collection periods. The goats were fed an isonitrogenous with low crude protein (~9.91%) but with incremental levels of metabolizable energy (ME), namely 7.01, 8.33, 9.66, and 10.98 MJ/kg DM, respectively. The detailed composition and nutrient analysis of the diets are provided in Supplementary Table S1. Goats were housed individually in metabolic cages, which allowed ad libitum access to the experimental diet, and the water was freely available throughout the experiment. The goats were fed twice daily at 08:00 and 16:30 throughout the experimental periods.

### 2.2. Fecal Sample Collection

Fecal samples of the experimental goats were collected rectally using sterile gloves before morning feeding on the last 3 days of each period. The fecal samples were mixed and then frozen in liquid nitrogen and stored at −80 °C for subsequent microbiome and metabolomic profiling analysis.

### 2.3. Microbiome Analysis

A commercial fecal DNA extraction kit (DP328, Tiangen Biotech, Beijing, China) was used to extract the total genomic DNA from the fecal samples, which was extracted from 1.00 g. The DNA quality was checked using a Thermo NanoDrop 2000 UV microphotometer and 1% agarose gel electrophoresis. The V3–V4 regions of the 16S rRNA gene were amplified with primers 338F (5′- ACTCCTACGGGAGGCAGCAG-3′) and 806R (5′- GGACTACHVGGGTWTCTAAT-3′). The bacterial 16S amplification and the quality filtering, clustering, and analysis of the 16S rRNA sequencing data were conducted in accordance with Liu et al.’s (2024) [15] approach. The reaction conditions and procedures of the PCR amplification of the 16S rRNA gene were as follows: initial denaturation at 95 °C for 3 min, followed by 30 cycles, including denaturation at 95 °C for 30 s, annealing at 55 °C for 30 s, and extension at 72 °C for 45 s, with a final extension at 72 °C for 10 min and holding at 10 °C until halter by user. The PCR mixtures were prepared in triplicate in 20 µL volumes, which consisted of 10 µL of 2×Pro Taq, 0.8 µL of forward primer (5 mM), 0.8 µL of reverse primer (5 mM), 10 ng/uL of template DNA, and ddH2O added until 20 µL was reached. Agarose gel (2.0%) electrophoresis (Axygen Biosciences, Union City, CA, USA) was applied to assess the success of PCR reactions. The PCR products were purified, quantified, and sequenced on the Illumina MiSeq PE300 platform (Illumina, San Diego, CA, USA; Majorbio Bio-Pharm Technology Co., Ltd., Shanghai, China). Data were analyzed using the free online Majorbio Cloud Platform (www.Majorbio.com, accessed on 25 February 2025).

### 2.4. Metabolomic Profiling

A total of 50.0 mg fecal sample of goats was added to a 2 mL centrifuge tube containing a 6 mm diameter grinding bead. Metabolites were extracted from 400 μL extraction solution, which consisted of methanol and water (*v*:*v* 4:1) and contained 0.02 mg/mL of L-2-chlorophenylalanine as the internal standard. The mixture samples were ground by a frozen tissue grinder (Wonbio-96c, Shanghai wanbo biotechnology Co., Ltd., Shanghai, China) for 6 min at −10 °C and 50 Hz, and then followed by low-temperature ultrasonic extraction for 30 min at 5 °C and 40 kHz. The samples were kept at −20 °C for 30 min and then centrifuged at 13,000 *g* for 15 min. The supernatant from each tube was transferred to the injection vial for LC-MS/MS analysis.

The LC-MS/MS analysis of the fecal sample was conducted on a SCIEX UPLC-Triple TOF 5600 system equipped with an ACQUITY HSS T3 column (100 mm × 2.1 mm, 1.8 μm; Waters, USA) at Majorbio Bio-Pharm Technology Co., Ltd. (Shanghai, China). The mobile phases consisted of solvent A (0.1% formic acid in water: acetonitrile; *v*/*v* 95:5) and solvent B (0.1% formic acid in acetonitrile/isopropanol/water; 47.5:47.5, *v*/*v*). The flow rate was maintained at 0.40 mL/min, and the column temperature was set at 40 °C.

The UPLC system was coupled to a quadrupole-time-of-flight mass spectrometer (Triple TOFTM5600+, Sciex, Boston, MA, USA) with an electrospray ionization (ESI) source operating in both positive mode and negative modes. The optimal conditions were set as follows: source temperature at 550 °C; curtain gas (CUR) at 30 psi; both Ion Source Gas1 and Gas2 at 50 psi; ion-spray voltage floating (ISVF) at −4000 V in negative mode and 5000 V in positive mode, respectively; declustering potential at 80 V; collision energy (CE), 20–60 eV rolling for MS/MS. Data acquisition was performed in the Information Dependent Acquisition (IDA) mode. The detection was carried out over a mass range of 50–1000 m/z.

Raw LC-MS data were preprocessed by Progenesis QI software (Waters Corporation, Milford, CT, USA), and then a three-dimensional data matrix in CSV format was exported. The information in this three-dimensional matrix included sample information, metabolite name, and mass spectral response intensity. Internal standard peaks, as well as any known false positive peaks (including noise, column bleed, and derivatized reagent peaks), were removed from the data matrix, de-redundified, and peak pooled. The metabolites were identified by searching databases, and the main databases were the HMDB (http://www.hmdb.ca/, accessed on 18 March 2025), Metlin (https://metlin.scripps.edu/, accessed on 18 March 2025), and the Majorbio Database.

The data were analyzed through the free online platform of the Majorbio cloud platform (cloud.majorbio.com, accessed on 18 March 2025). Metabolic features detected ≥80% of samples were retained. After filtering, minimum metabolite values were imputed for specific samples in which the metabolite levels fell below the lower limit of quantitation, and each metabolic feature was normalized by sum. To minimize the errors caused by sample preparation and instrument instability, the response intensity of the sample mass spectrum peaks was normalized by the sum normalization method, and then the normalized data matrix was obtained. Meanwhile, variables with relative standard deviation (RSD) >30% of QC samples were excluded. The normalized matrix was log10-transformed for subsequent analysis.

### 2.5. Statistical Analysis

The data of alpha diversity, the relative abundance of the phylum, and genus (>0.50%) of fecal bacteria communities were analyzed using the mixed model procedure of the SAS statistical package (SAS version 9.4, SAS Inst. Inc., Cary, NC, USA). The model was Yijkl = μ + Si + Rj + Ck + Tl + ϵijkl, where YijklYijkl = observed response for the i-th square, j-th row, k-th column, and l-th treatment; μ = overall mean; Si = effect of the i-th square (i = 1, 2); Rj = effect of the j-th row within the square (j = 1, 2, 3, 4); Ck = effect of the k-th column within the square (k = 1, 2, 3, 4); Tl = effect of the l-th treatment (metabolites energy levels; 7.01, 8.33, 9.66, and 10.98 MJ/kg DM); and ϵijkl = random error associated with the observation. Polynomial contrasts were used to determine whether the effects of dietary energy levels on the measured variables were linear or quadratic. Data were expressed as mean ± SEM. Pearson’s correlation analysis linked microbial taxa with metabolites using the Origin (version 2023) and GraphPad Prism (version 7, Boston, MA, USA). Statistical significance was set at *p* < 0.05.

## 3. Results

### 3.1. Overall Structure of Fecal Bacterial Communities

The 2,354,776 raw reads were generated from the 32 fecal samples, and 2,302,736 high-quality sequences remained after quality filtering and removal of chimeric sequences. The 6660 OTUs were obtained based on 97% nucleotide sequence identity analysis among reads.

**Figure 1:**
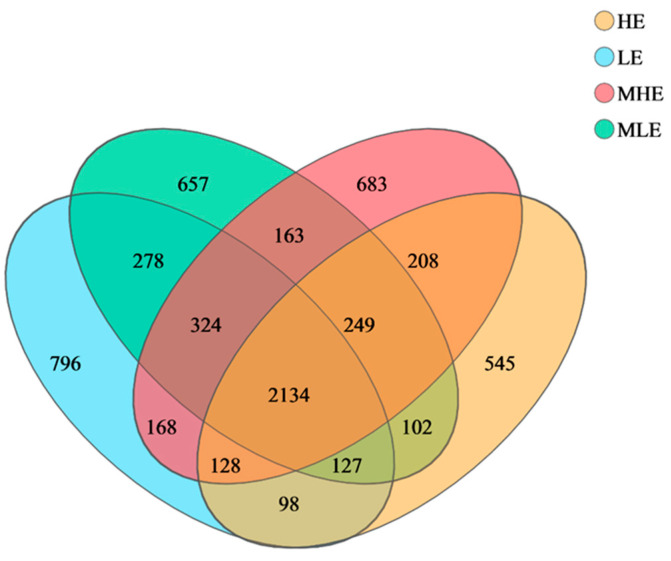
Veen diagrams of OTUs in the feces of goats when consuming a lower-protein diet with varying energy levels. LE, low metabolizable energy (=7.01 MJ/kg DM) group; MLE, middle–low metabolizable energy (=8.33 MJ/kg DM) group; MHE, middle–high metabolizable energy (=9.66 MJ/kg DM) group; HE, high metabolizable energy (=10.98 MJ/kg DM) group. n = 8 samples per group.

The 2134 OTUs were shared among the four treatment groups in the 32 fecal samples, which take up 52.7%, 52.9%, 52.6%, and 59.4% of the total OTUs in LE, MLE, MHE, and HE groups, respectively (Figure 1). Additionally, the specific number of OTUs in the LE, MLE, MHE, and HE groups were 796, 657, 683, and 545, respectively. The Sobs, Shannon, Simpson, ACE, Chao, and coverage indices of fecal bacteria did not differ among the four groups (Table 1).

Energy is the effect of dietary metabolizable energy levels; Energy-L is the linear effect of dietary metabolizable energy levels; Energy-Q is the quadratic effect of dietary metabolizable energy levels.

### 3.2. Analysis of Composition and Difference of Microbiota

**Figure 2:**
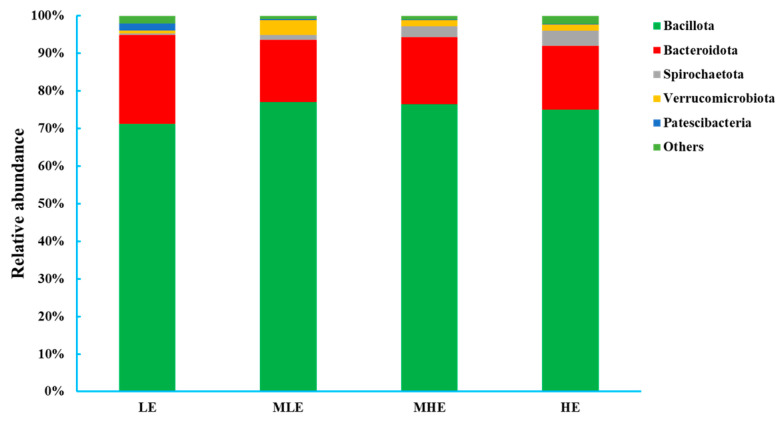
Relative abundances of bacterial phyla (>0.50% of total reads) in the feces of goats when consuming a lower-protein diet with varying energy levels. LE, low metabolizable energy (=7.01 MJ/kg DM) group; MLE, middle–low metabolizable energy (=8.33 MJ/kg DM) group; MHE, middle–high metabolizable energy (=9.66 MJ/kg DM) group; HE, high metabolizable energy (=10.98 MJ/kg DM) group. n = 8 samples per group.

At the phylum level, 18 bacteria were identified in the 32 fecal samples. The dominant phylum was Firmicutes with 71.2%, 77.1%, 76.6%, and 75.0% in the LE, MLE, MHE, and HE groups, respectively, and then followed by Bacteroidetes with 23.6%, 16.5%, 17.8%, and 17.0% in the LE, MLE, MHE, and HE groups, respectively (Figure 2; Supplementary Table S1). The relative abundances of Firmicutes and Spirochaetote increased linearly, whereas the Bacteroidota and Patescibacteria decreased linearly with increasing dietary energy levels (*p* < 0.05). The relative abundances of Verrucomicrobiota increased quadratically, whereas others decreased quadratically with increasing dietary energy levels (*p* < 0.05).

**Figure 3:**
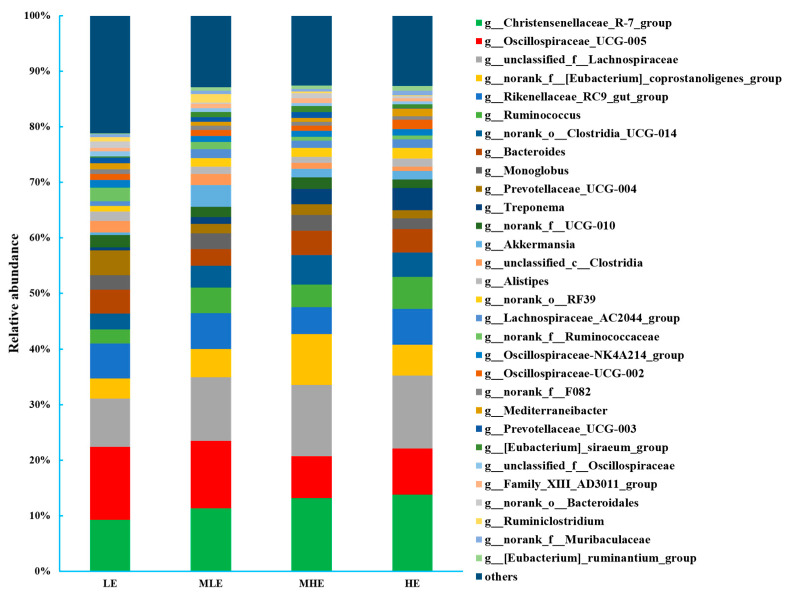
Bacterial composition at the genus level (>0.50% total reads) in feces of goats when consuming a lower-protein diet with varying energy levels. LE, low metabolizable energy (=7.01 MJ/kg DM) group; MLE, middle–low metabolizable energy (=8.33 MJ/kg DM) group; MHE, middle–high metabolizable energy (=9.66 MJ/kg DM) group; HE, high metabolizable energy (=10.98 MJ/kg DM) group. n = 8 samples per group.

At the genus level, a total of 316 bacteria were identified in the 32 fecal samples. The dominant genus was *Christensenellaceae_R-7_group* with 9.28%, 11.34%, 13.2%, and 13.8% in the LE, MLE, MHE, and HE groups, respectively, and the second most abundant genus was *unclassified_f__Lachnospiraceae* with 8.68%, 11.5%, 12.9%, and 13.2% in the LE, MLE, MHE, and HE groups, respectively (Figure 3; Supplementary Table S2). The relative abundances of *Christensenellaceae_R-7_group*, *unclassified_f__Lachnospiraceae*, *Ruminococcus*, *norank_o__Clostridia_UCG-014*, *Treponema*, *[Eubacterium]_siraeum_group*, and *[Eubacterium]_ruminantium_group* increased linearly, whereas the relative abundance of *Oscillospiraceae_UCG-005*, *norank_f__[Eubacterium]_coprostanoligenes_group*, *Prevotellaceae_UCG-004*, *unclassified_c__Clostridia*, *norank_f__Ruminococcaceae, unclassified_f__Oscillospiraceae*, and others decreased linearly with an increasing dietary energy levels (*p* < 0.05).

**Figure 4:**
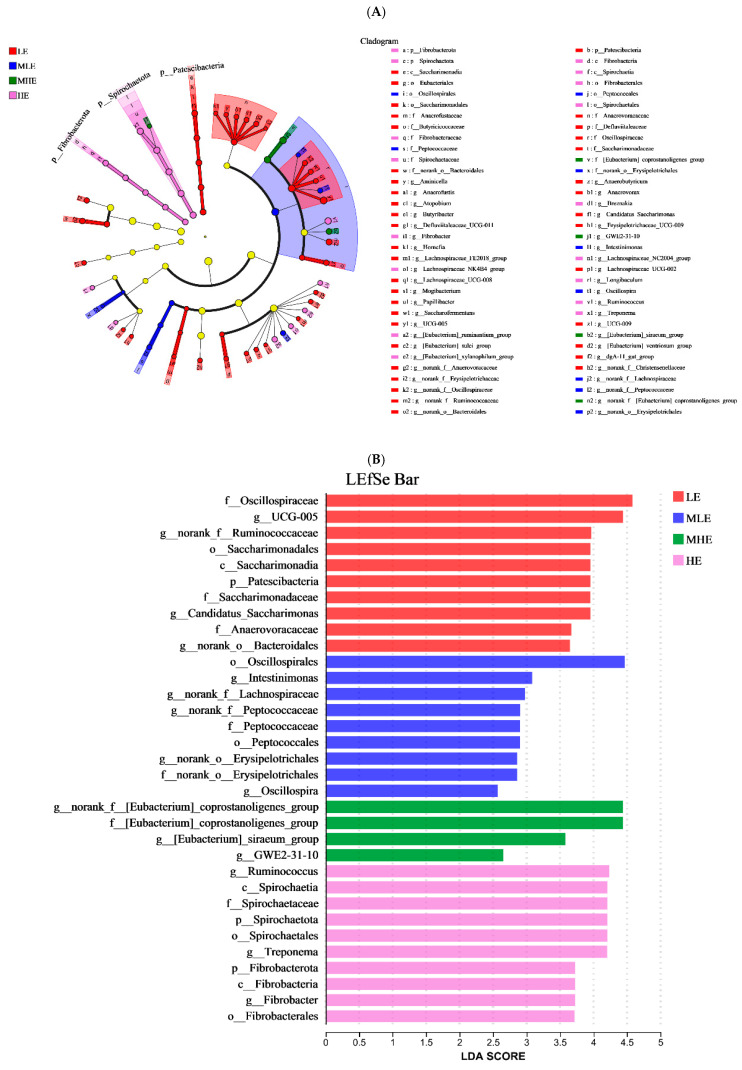
Linear discriminant analysis effect size (LEfSe) analysis: (**A**) The cladogram diagram shows the microbial species with significant differences among the 4 groups. (**B**) Species with significant differences that have an LDA score greater than the estimated value; the default score is 2.0. The length of the histogram represents the LDA score; prefixes represent abbreviations for the taxonomic rank of each taxon, phylum (p_), class (c_), order (o_), family (f_), and genus (g_). LE, low metabolizable energy (=7.01 MJ/kg DM) group; MLE, middle–low metabolizable energy (=8.33 MJ/kg DM) group; MHE, middle–high metabolizable energy (=9.66 MJ/kg DM) group; HE, high metabolizable energy (=10.98 MJ/kg DM) group. n = 8 samples per group.

The LEfSe analysis was performed to identify microbial taxa that serve as biomarkers among the four groups (Figure 4A,B). The f_Oscillospiraceae, *g_UCG-005*, *g_norank_f_Ruminococcaceae*, o_Saccharimonadales, c_Saccarimonaadia, p_Patescibacteria, f_Saccarimonadaceae, *g_Candidatus_Saccharimonas*, f_Anaerovoracaceae, and *g_norank_o_Bacteroidales* remarkably enriched in the LE groups. The o_Oscillospirales, g_Intestinimonas, *g_norank_f_Lachnospiraceae*, *g_norank_f_Peptococcaceae*, f_Peptococcaceae, o_Petococcaceae, *g_norank_o_Erysipelotrichales*, f_norank_o_Erysipelotrichales, and *g_Oscillospira* remarkably enriched in the MLE group. The *g_norank_f_[Eubacterium]_coprostanoli- genes_group*, f_[Eubacterium]_coprostanoligenes_group, *g_[Eubacterium]_siraeum_ group*, and *g_GWE2-31-10* remarkably enriched in the MHE group. The *g_Ruminococcus*, c-Spirochaetia, f_Spirochaetaceae, p_Spirochaetales, *g_Treponema*, p_Fibrobacterota, c-Fibrobacterota, *g_Fibrobacter*, and o_Fibrobacterales remarkably enriched in the HE group.

### 3.3. Analysis of Differential Metabolites

**Figure 5:**
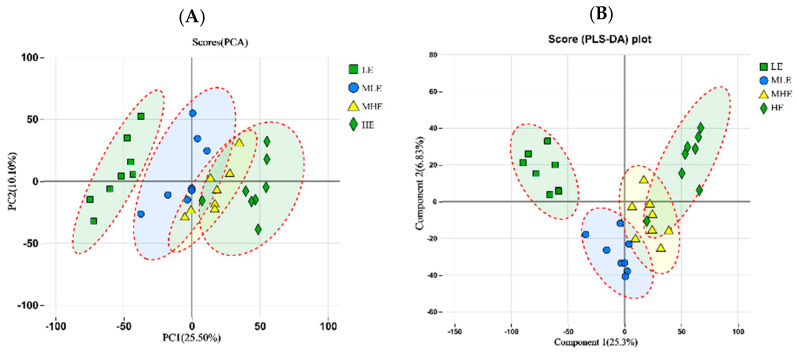
The principle component analysis (PCA); (**A**) and partial least squares discrimination analysis (PLS-DA); (**B**) of metabolic composition on fecal samples in goats when consuming a lower-protein diet with varying energy levels. LE, low metabolizable energy (=7.01 MJ/kg DM) group; MLE, middle–low metabolizable energy (=8.33 MJ/kg DM) group; MHE, middle–high metabolizable energy (=9.66 MJ/kg DM) group; HE, high metabolizable energy (=10.98 MJ/kg DM) group. n = 8 samples per group.

The principal component analysis (PCA) showed that LE, MLE, MHE, and HE groups could be distinguished, indicating that different treatments change the metabolite diversity of the feces of goats as a whole (Figure 5A). However, the partial least squares discriminant analysis (PLS-DA) model showed significant differences among the four groups (Figure 5B).

**Figure 6:**
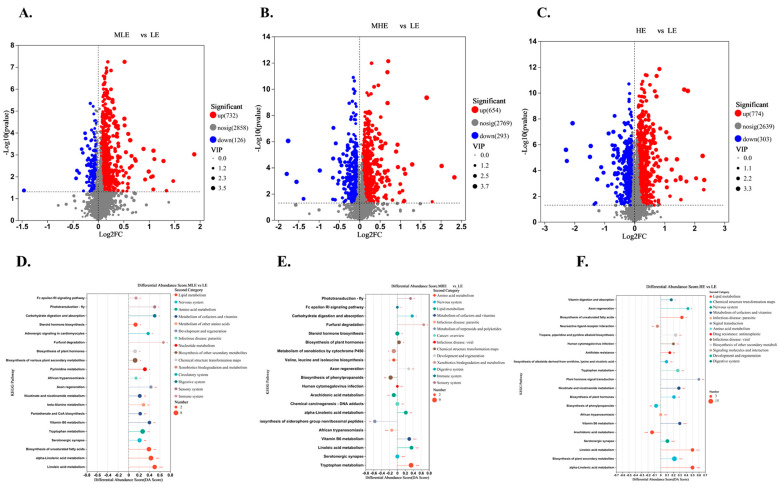
Effect of fecal metabolites of goats when consuming a lower-protein diet with varying energy levels: (**A**,**D**) Results of differential metabolites and metabolic pathway analysis were presented in feces between LE and MLE groups. (**B**,**E**) Results of differential metabolites and metabolic pathway analysis were presented in feces between LE and MHE groups. (**C**,**F**) Results of differential metabolites and metabolic pathway analysis were presented in feces between LE and HE groups. The figure shows the relative content changes in differential metabolites in the form of bar charts. Upregulated metabolites are represented by red bars, and down-regulated metabolites are represented by blue bars. LE, low metabolizable energy (=7.01 MJ/kg DM) group; MLE, middle–low metabolizable energy (=8.33 MJ/kg DM) group; MHE, middle–high metabolizable energy (=9.66 MJ/kg DM) group; HE, high metabolizable energy (=10.98 MJ/kg DM) group. n = 8 samples per group.

The results of the volcano plot analysis showed that the fecal metabolites of the MLE, MHE, and HE groups compared to the LE group were quite different. The total number of differential metabolites between MLE and LE groups was 858, of which 732 were upregulated, and 126 were down-regulated (Figure 6A). The total number of differential metabolites between MHE and LE groups was 947, of which 654 were upregulated, and 293 were down-regulated (Figure 6B). The total number of differential metabolites between HE and LE groups was 1007, of which 774 were upregulated, and 303 were down-regulated (Figure 6C).

The KEGG enrichment analysis was performed on differential metabolites for fecal samples of goats when consuming a lower-protein diet with varying energy levels. It was found that metabolites from LE and MLE fecal samples were significantly enriched in pathways related to steroid hormone biosynthesis, biosynthesis of various plant secondary metabolites, tryptophan metabolism, biosynthesis of unsaturated fatty acids, alpha-linolenic acid metabolism, linoleic acid metabolism (Figure 6D). The metabolites were significantly enriched in pathways related to tryptophan metabolism, vitamin B6 metabolism, linoleic acid metabolism, and steroid hormone biosynthesis between MHE and LE groups (Figure 6E). The metabolites were significantly enriched in pathways related to biosynthesis of plant secondary metabolites, alpha-linolenic acid metabolism, linoleic acid metabolism, and biosynthesis of unsaturated fatty acids between HE and LE groups (Figure 6F). In addition, the lipid metabolism, for example, the alpha-linolenic acid metabolism, linoleic acid metabolism, fatty acid biosynthesis, and biosynthesis of unsaturated fatty acids was greater in the MLE, MHE, and HE groups than in the LE group (Supplementary File S2). The amino acids, for example, the valine, leucine and isoleucine biosynthesis, lysine biosynthesis, arginine and proline metabolism, tyrosine metabolism, and tryptophan metabolism were greater, whereas the arginine biosynthesis, alanine, aspartate and glutamate metabolism, glycine, serine and threonine metabolism, histidine metabolism, and cysteine and methionine metabolism were lesser in MLE, MHE, and HE groups than in the LE group (Supplementary File S2).

### 3.4. Pearson Correlation Between Fecal Bacterial Communities (at Genus Level) and Metabolomics

**Figure 7:**
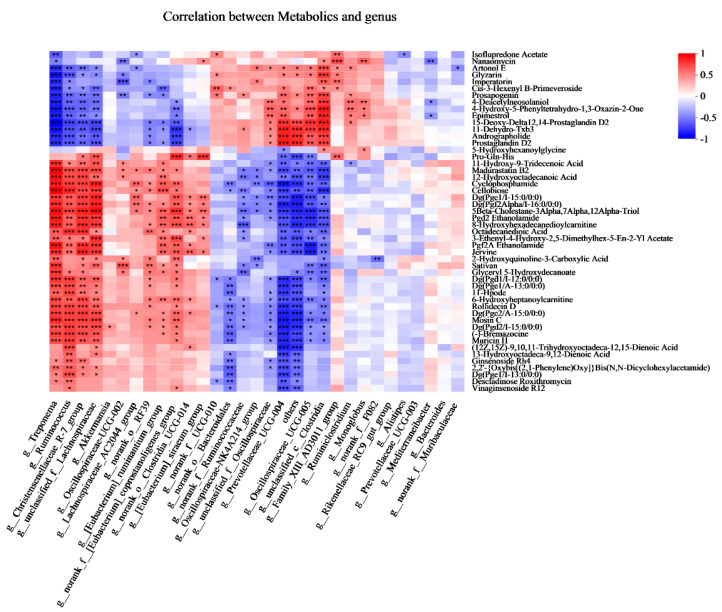
Correlations analysis between the fecal bacteria (at genus) and fecal metabolites (Top 50) based on Pearson’s rank correlation analysis. * *p* < 0.05, ** *p* < 0.01, and *** *p* < 0.001 according to Pearson’s rank correlation coefficient.

To explore the functional correlation between the fecal bacterial community’s changes and metabolite perturbations, a Pearson correlation matrix was generated by calculating the Pearson correlation coefficient (Figure 7). More interestingly, the cellobiose was positively associated with *Treponema*, *Ruminococcus*, *Christensenellaceae_R-7_group*, *unclassified_f_Lachnospiraceae*, *Lachnospiraceae_AC2044_group*, *norank_o_RF-039*, *[Eubacterium]_ruminantium_group*, and *norank_f__[Eubacterium]_ coprostanoligenes_group*, whereas it was negatively associated with *norank_o__ Bacteroidales*, *norank_f__Ruminococcaceae*, *Oscillospiraceae-NK4A214_group*, *unclassified_f__Oscillospiraceae*, *Prevotellaceae_UCG-004*, *others*, *Oscillospiraceae _UCG-005*, *unclassified_c__Clostridia*. The Pro-Gln-His was positively associated with *Christensenellaceae_R-7_group*, *unclassified_f__Lachnospiraceae*, *Akkermansia*, *Oscillospiraceae-UCG-002*, *Lachnospiraceae_AC2044_group*, *norank_o__RF39*, *[Eubacterium]_ruminantium_group*, *norank_f__[Eubacterium]_Coprostanoligenes _group*, *norank_o__Clostridia_UCG-014*, *[Eubacterium]_ siraeum_group*, and negatively associated with *Prevotellaceae_UCG-004*, others, *Oscillospiraceae_UCG-005*, *Family_XIII_AD3011_group*.

## 4. Discussion

The present study utilized the Latin square design in animal nutrition, but it had a limited sample size. However, we recognize that the absence of formal power calculations or supporting physiological data may affect the generalizability of our findings, particularly given the inherent variability of microbiome and metabolomic datasets. Future work would benefit from integrating biochemical parameters (e.g., growth performance, plasma metabolites) and prospective power analyses to strengthen causal inferences.

Our results showed that the two most predominant phyla in the fecal samples of the goats were Bacteroidetes and Firmicutes, which is in agreement with previous studies in fecal samples of goats [15], cattle [16], dairy cows [17]. In the present study, the types of the dominant microbiota in fecal samples of the goats were similar, whereas the relative abundance of Firmicutes, Bacteroidota, Spirochaetota, Verrucomicrobiota, Patescibacteria, and others was different among the four groups. This indicates that the fecal microbial communities of goats were relatively stable, and dietary energy levels had no significant effect on the types, whereas they could significantly change the proportion of the dominant microbial community in the feces. The relative abundance of Firmicutes was greater than Bacteroidota, which could be explained by the fact that Firmicutes composed a greater amount of the microbial population in the feces and was followed by Bacteroidota [18]. Our results also reported that the relative abundance of Firmicutes increased and Bacteroidetes decreased in fecal samples of goats when consuming a lower-protein diet with an increasing energy level, which was in agreement with previous reports in the rumen samples of yaks [19] and goats [20]. The Spirochaetota mainly relies on the hydrolysis of complex polysaccharides in the plant cell wall. In the present study, our results showed that the relative abundance of Spirochaetota was increased with increasing dietary energy levels, which is in agreement with a previous study in fecal samples of dairy cows when consuming a diet with energy levels increased (7.03 vs. 7.70 MJ/kg) [21].

In the present study, the *Christensenellaceae_R-7_group* and *unclassified_f__Lachnospiraceae* were the top two genera. Previous studies reported that the predominant genera in fecal samples were *Rikenellaceae_RC9_gut_group* and *Ruminococcaceae_UCG-005* in Boer goats [22], *UCG-005* and *Christensenellaceae_R-7_group* in Qianbei goats, and the *unclassified_f_Lachnospiraceae* and *Oscillospiraceae-UCG-005* in Leizhou goats [15]. This difference may be related to the differences in animal breeds and dietary composition among these studies. Chen et al. [23] reported that *Lachnospiraceae* could potentially serve as probiotics, which could enhance fermentation efficiency, promote host health, and correlate positively with ADG. In addition, numerous studies also reported that average daily gain could increase with increasing dietary energy levels in Boer goats [2], Yunnan semi-fine wool sheep [3], fattening Angus steers [23], and yaks [24]. Hence, this could explain why the relative abundance of *unclassified_f__Lachnospiraceae* was increased with the dietary energy levels. *Akkermansia*, a relevant mucin degrader from the vertebrate gut microbiota, is a member of the deeply branched Verrucomicrobiota, as well as the only known member of this phylum to be described as inhabitants of the gut [25]. The relative abundance of *Akkermansia* was generally decreased with inflammatory bowel disease in mouse models [26]. In this study, we found that the relative abundance of *Akkermansia* was lower in the LE group. A previous study reported that the abundance of *Christensenellaceae_R-7_group*, *NK4A214_group*, *Ruminococcus*, *norank_f__Eubacterium_ coprostanoligenes_group*, *norank_f__norank_o__Clostridia_UCG-014*, *Lachnospiraceae_ NK3A20_group*, *Acetitomaculum*, and *Family_XIII_AD3011_group* increased with increasing concentrate supplementation, while the relative abundance of *Rikenellaceae_RC9_gut_ group* decreased [27]. In the present study, the relative abundance of *Christensenellaceae_R-7_group*, *Ruminococcus*, *norank_f__[Eubacterium]_coprostanoligenes _group*, and *norank_f__norank_o__Clostridia_ UCG-014* increased, whereas the abundance of *Rikenellaceae_RC9_gut_ group* decreased with increasing dietary energy levels, which partly in agreement with the results from Yi et al. [27]. A previous study reported that *Ruminococcus* possesses activities that break down cellulose and hemicellulose, resulting in the production of acetic acid, butyric acid, formate, and hydrogen [28]. In the present study, the relative abundance of *Ruminococcus* increased with increasing dietary energy levels, which is in agreement with a previous study reported that *Ruminococcus* played a regulatory role in maintaining the stability of the rumen endo-environment in yaks when the dietary energy levels increased [19]. The *uncassified Clostridia* and *unclassified_f__Lachnospiraceae* refer to microbiota that have not yet been classified or identified and have various health effects. The ecological or functional role of these genera needs to be clarified in further study.

Metabolomics data can provide a greater insight into the influence of diet on the body. In the present study, the lipid metabolism, for example, the alpha-linolenic acid metabolism, linoleic acid metabolism, fatty acid biosynthesis, and biosynthesis of unsaturated fatty acids was greater in the MLE, MHE, and HE groups than in the LE group. These could be explained by increasing dietary energy levels. Amino acids are essential nutrients for the body. The amino acid metabolism also changed among the four groups. Interestingly, we found that the valine, leucine, and isoleucine biosynthesis were greater with increasing dietary energy levels, which is in agreement with a previous study in yaks and cattle [19].

In the present study, we found that the *Christensenellaceae_R-7_group* was positively associated with the Pro-Gln-His. This could be explained by the abundance of *Lachnospiraceae, which* was positively correlated with the metabolism of amino acids [29,30]. A previous study reported that the *Rikenellaceae_RC9_gut_group* has played an important role in modulating meat amino acid; in particular, it was positively correlated with the amino acid content of histidine in Boer Crossbred goats [31]. Interestingly, in the present study, the results showed that there was no correlation between *Rikenellaceae_RC9_gut_group* and Pro-Gln-His. We inferred that this difference could be explained by the levels of dietary protein levels. *Christensenellaceae* was regarded as a fibrolytic bacteria, which produce α-arabinosidase, β-galactosidase, and β-glucosidase [32,33]. *Ruminococcus* is also regarded as a cellulolytic bacterium that can produce acetic acid, formic acid, ethanol, and lactic acid [19]. The members of *Lachnospiraceae*, for example, *unclassified_f__Lachnospiraceae* and *Lachnospiraceae_AC2044_group*, could degrade plant cellulose and hemicellulose. Cellobiose is a disaccharide of two glucose units linked by a β-1,4ʹ-glycosidic bond. In the present study, we found that the cellobiose was positively associated with *Ruminococcus*, *Christensenellaceae_R-7_group*, *unclassified_f_Lachnospiraceae*, *Lachnospiraceae_AC2044_group*. Furthermore, more information is needed to clarify the function of these genera.

## 5. Conclusions

The microbial composition and metabolites of feces were found to be altered in goats when consuming a lower-protein diet with varying energy levels. At the genus level, the relative abundances of *Christensenellaceae_R-7_group*, *unclassified_f__Lachnosp-iraceae*, *Ruminococcus*, *norank_o__Clostridia_UCG-014*, *Treponema*, *[Eubacterium] _siraeum_group*, and *[Eubacterium]_ruminantium_group* increased linearly, whereas the *Oscillospiraceae_UCG-005*, *norank_f__[Eubacterium] _coprostanoligenes_group*, *Prevotellaceae_UCG-004*, *unclassified_c__Clostridia*, *norank_f__Ruminococcaceae*, *unclassified_f__Oscillospiraceae*, and others decreased linearly with an increasing dietary energy levels. In addition, the amino acids, for example, valine, leucine, and isoleucine biosynthesis, and lipid metabolism, for example, the alpha-linolenic acid metabolism, linoleic acid metabolism, fatty acid biosynthesis, and biosynthesis of unsaturated fatty acids, was greater in the MLE, MHE, and HE groups than in the LE group. This study provides further information regarding the effects of fecal bacterial community composition and metabolites in goats when consuming a lower-protein diet with different energy levels. In the future, more information is needed on growth performance, serum index, welfare, and the relationship between the bacterial communities and metabolite needs in goats when consuming a lower-protein diet with different energy levels.

## Figures and Tables

**Table 1:** The alpha diversity in the feces of goats when consuming a lower-protein diet with varying energy levels.

Items	Dietary Energy Levels				SEM	*p*-Values		
	LE	MLE	MHE	HE		Energy	Energy-L	Energy-Q
Sobs	1556	1585	1610	1380	47.0	0.310	0.236	0.176
Shannon	5.33	5.29	5.43	5.19	0.053	0.485	0.581	0.355
Simpson	0.018	0.022	0.014	0.017	0.0017	0.322	0.450	0.950
Ace	1936	2013	2049	1733	62.6	0.290	0.308	0.123
Chao	1886	1964	1999	1697	60.1	0.297	0.326	0.121
Coverage	0.990	0.989	0.989	0.991	0.0004	0.290	0.430	0.091

SEM, standard error of the mean; LE, low metabolizable energy (=7.01 MJ/kg DM) group; MLE, middle–low metabolizable energy (=8.33 MJ/kg DM) group; MHE, middle–high metabolizable energy (=9.66 MJ/kg DM) group; HE, high metabolizable energy (=10.98 MJ/kg DM) group. n = 8 samples per group.

## Data Availability

The data used are confidential and will be made available upon request from the corresponding author.

## Supplementary Materials

The following supporting information can be downloaded at: https://www.mdpi.com/article/10.3390/microorganisms13040941/s1, Table S1: Dietary ingredients and chemical composition of the experiment; Table S2: Fecal bacterial relative abundances (at phylum level, >0.05% of total reads) in goats in response to lower protein dietary with different energy levels; Table S3: Fecal bacterial relative abundances (at genus level, >0.05% of total reads) in goats in response to lower protein dietary with different energy levels.
