# Supplementary material for: Analysis of Fecal Microbiome and Metabolome Changes in Goats When Consuming a Lower-Protein Diet with Varying Energy Levels
Source: Microorganisms. 2025 Apr 18;13(4):941. doi: 10.3390/microorganisms13040941 (PMC12029318; doi:10.3390/microorganisms13040941)
Supplement: Supplementary file 1 [file microorganisms-13-00941-s001.zip › supplementary file 1 diet and bacteria.pdf]

Table S1 Dietary ingredients and chemical composition of the experiment.

Items	LE	MLE	MHE	HE
Ingredient, % of DM				
Oat hull	50.0	47.0	43.0	29.0
Rice hull meal	30.0	18.0	7.00	6.00
Corn, ground	1.70	7.10	14.05	23.95
Soybean meal	10.5	7.60	4.00	2.50
Wheat bran	1.60	8.00	16.00	22.00
Soybean hulls	2.00	5.90	7.45	4.10
Palm meal	0.10	1.00	3.10	5.65
Soybean oil	0.10	1.40	1.40	2.80
Commercial Premix[1]	4.00	4.00	4.00	4.00
Nutrient composition, % of DM				
Dry matter	93.2	94.6	96.8	93.4
Crude protein	9.91	9.88	9.90	9.93
Ether extract	3.88	5.03	5.33	6.72
Neutral detergent fiber	71.1	66.2	57.6	48.0
Acid detergent fiber	30.1	25.5	22.0	19.6
Metabolizable energy	7.01	8.33	9.66	10.98

LE, low metabolizable energy (=7.01 MJ/kg DM) group; MLE, middle-low metabolizable energy (=8.33 MJ/kg DM) group; MHE, middle high metabolizable energy (9.66 MJ/kg DM) group; HE, high metabolizable energy (10.98 MJ/kg) group.

[1] Premix (per kg) contains: Cu 250 mg, Fe 1,250 mg, Zn 1,000 mg, Mn 1050 mg, I 30 mg, Se 30 mg, Co 50 mg, Vitamin A 100 kIU, Vitamin D3 4,500 IU, Vitamin E 900 mg, Vitamin K3 45 mg.

Table S2 Fecal bacterial relative abundances (at phylum level, > 0.05% of total reads) in goats in response to lower protein dietary with different energy levels

Items	Dietary energy levels				SEM	P-values		
	LE	MLE	MHE	HE		E	E-L	E-Q
Firmicutes	71.2	77.1	76.6	75.0	0.84	0.046	0.022	0.021
Bacteroidota	23.6	16.5	17.8	17.0	0.71	<0.001	<0.001	0.005
Spirochaetota	0.53	1.22	2.84	4.04	0.271	<0.001	<0.001	0.305
Verrucomicrobiota	0.71	3.96	1.62	1.63	0.415	0.029	0.904	0.039
Patescibacteria	1.79	0.32	0.16	0.08	0.153	<0.001	<0.001	0.001
Others	2.16	0.92	1.08	2.26	0.174	0.003	0.707	<0.001

SEM, standard error of the mean; LE, low metabolizable energy (=7.01 MJ/kg DM) group; MLE, middle-low metabolizable energy (=8.33 MJ/kg DM) group; MHE, middle high metabolizable energy (9.66 MJ/kg DM) group; HE, high metabolizable energy (10.98 MJ/kg) group. n=8. Energy, the effect of dietary metabolizable energy levels; Energy-L, Linear effect of dietary metabolizable energy levels; Energy-Q, Quadratic effect of dietary metabolizable energy levels.

Table S3 Fecal bacterial relative abundances (at genus level, > 0.05% of total reads) in goats in response to lower protein dietary with different energy levels

Items	Dietary energy levels				SEM	P-values		
	LE	MLE	MHE	HE		E	E-L	E-Q
g__Christensenellaceae_R-7_group	9.28	11.3	13.2	13.8	0.46	<0.001	<0.001	0.334
g__unclassified_f__Lachnospiraceae	8.68	11.5	12.9	13.2	0.44	<0.001	<0.001	0.064
g__Oscillospiraceae_UCG-005	13.1	12.1	7.56	8.24	0.56	<0.001	<0.001	0.262
g__norank_f__[Eubacterium]_coprostanoligenes_group	3.57	5.09	9.11	5.58	0.381	<0.001	<0.001	<0.001
g__Rikenellaceae_RC9_gut_group	6.30	6.44	4.84	6.43	0.160	<0.001	0.228	0.003
g__Ruminococcus	2.53	4.62	4.06	5.76	0.270	<0.001	<0.001	0.596
g__norank_o__Clostridia_UCG-014	2.91	3.91	5.35	4.36	0.324	0.053	0.039	0.106
g__Bacteroides	4.28	3.01	4.33	4.25	0.241	0.148	0.550	0.209
g__Monoglobus	2.58	2.82	2.86	1.95	0.165	0.176	0.204	0.079
g__Prevotellaceae_UCG-004	4.49	1.74	1.91	1.45	0.270	<0.001	<0.001	0.002
g__Treponema	0.52	1.21	2.80	4.00	0.275	<0.001	<0.001	0.351
g__norank_f__UCG-010	2.22	1.87	2.06	1.51	0.129	0.253	0.101	0.693
g__Akkermansia	0.50	3.88	1.51	1.55	0.342	0.001	0.749	0.005
g__unclassified_c__Clostridia	2.08	2.01	1.09	0.78	0.134	<0.001	0.000	0.510
g__Alistipes	1.68	1.32	1.06	1.48	0.188	0.709	0.626	0.318
g__norank_o__RF39	0.99	1.56	1.63	1.90	0.158	0.222	0.051	0.617
g__Lachnospiraceae_AC2044_group	0.85	1.61	1.31	1.51	0.124	0.127	0.116	0.249
g__norank_f__Ruminococcaceae	2.46	1.27	0.69	0.72	0.317	0.016	0.042	0.326
g__Oscillospiraceae-NK4A214_group	1.37	1.06	1.10	1.10	0.058	0.201	0.139	0.165
g__Oscillospiraceae-UCG-002	1.07	1.09	0.93	1.71	0.150	0.260	0.191	0.201
g__norank_f__F082	0.88	0.75	0.71	0.66	0.122	0.933	0.534	0.872
g__Mediterraneibacter	1.09	0.69	0.62	1.37	0.158	0.315	0.588	0.080
g__Prevotellaceae_UCG-003	0.94	0.90	1.09	0.06	0.398	0.808	0.508	0.550
g__[Eubacterium]_siraeum_group	0.29	0.93	1.09	0.71	0.091	0.007	0.047	0.003
g__unclassified_f__Oscillospiraceae	0.91	0.70	0.55	0.53	0.059	0.077	0.014	0.402
g__Family_XIII_AD3011_group	0.60	0.72	0.84	0.53	0.053	0.190	0.862	0.051
g__norank_o__Bacteroidales	1.13	0.23	0.96	0.38	0.127	0.023	0.141	0.492
g__Ruminiclostridium	0.77	1.57	0.29	0.15	0.224	0.095	0.104	0.274
g__norank_f__Muribaculaceae	0.52	0.60	0.47	0.87	0.133	0.737	0.461	0.556
g__[Eubacterium]_ruminantium_group	0.18	0.63	0.60	0.83	0.085	0.042	0.010	0.487
others	21.17	12.85	12.56	12.65	0.81	<0.001	<0.001	<0.001

SEM, standard error of the mean; LE, low metabolizable energy (=7.01 MJ/kg DM) group; MLE, middle-low metabolizable energy (=8.33 MJ/kg DM) group; MHE, middle high metabolizable energy (9.66 MJ/kg DM) group; HE, high metabolizable energy (10.98 MJ/kg) group. n=8. Energy, the effect of dietary metabolizable energy levels; Energy-L, Linear effect of dietary metabolizable energy levels; Energy-Q, Quadratic effect of dietary metabolizable energy levels.
